# Enhancing Monkeypox Diagnosis with Transformers: Bridging Explainability and Performance with Quantitative Validation

**DOI:** 10.3390/diagnostics15182354

**Published:** 2025-09-16

**Authors:** Delal Şeker, Abdulnasır Yıldız

**Affiliations:** Department of Electrical and Electronics Engineering, Dicle University, Diyarbakir 21280, Turkey; abnayil@dicle.edu.tr

**Keywords:** monkeypox, Transformer models, explainable artificial intelligence, hybrid heatmap, causal metrics

## Abstract

**Background/Objectives**: Monkeypox is a zoonotic virus that presents with smallpox-like symptoms, making visual diagnosis challenging due to overlap with other dermatological conditions. Existing AI-based studies on monkeypox classification have largely relied on Convolutional Neural Networks (CNNs), with limited exploration of Transformer architectures or robust interpretability frameworks. Moreover, most explainability research still depends on conventional heatmap techniques without systematic evaluation. This study addresses these gaps by applying Transformer-based models and introducing a novel hybrid explainability approach. **Methods**: We fine-tuned Vision Transformer (ViT) and Data-Efficient Image Transformer (DeiT) models for both binary and multi-class classification of monkeypox and other skin lesions. To improve interpretability, we integrated multiple explainable AI techniques—Gradient-weighted Class Activation Mapping (Grad-CAM), Layer-wise Relevance Propagation (LRP), and Attention Rollout (AR)—and proposed a hybrid method that combines these heatmaps using Principal Component Analysis (PCA). The reliability of explanations was quantitatively assessed using deletion and insertion metrics. **Results**: ViT achieved superior performance with an AUC of 0.9192 in binary classification and 0.9784 in multi-class tasks, outperforming DeiT. The hybrid approach (Grad-CAM + LRP) produced the most informative explanations, achieving higher insertion scores and lower deletion scores than individual methods, thereby enhancing clinical reliability. **Conclusions**: This study is among the first to combine Transformer models with systematically evaluated hybrid explainability techniques for monkeypox classification. By improving both predictive performance and interpretability, our framework contributes to more transparent and clinically relevant AI applications in dermatology. Future work should expand datasets and integrate clinical metadata to further improve generalizability.

## 1. Introduction

Monkeypox is a zoonotic viral infection first identified in humans in the latter half of the 20th century. It is caused by a virus from the *Poxviridae* family and exhibits clinical similarities to smallpox, albeit with generally milder symptoms. Human infection occurs through direct contact with infected animals, their bodily fluids, or contaminated materials. Additionally, human infection occurs through direct contact with skin lesions, or shared use of contaminated objects [1]. The disease typically manifests with fever, lymphadenopathy, myalgia, and characteristic skin rashes that may resemble those of chickenpox or other vesicular skin conditions, complicating differential diagnosis. Although monkeypox is often self-limiting, severe complications can arise in immunocompromised individuals and children [2]. Recent global outbreaks have highlighted the urgent need for improved diagnostic and therapeutic strategies. Current diagnostic methods primarily rely on clinical evaluation and molecular testing (e.g., PCR); however, visual assessment of skin lesions remains subjective and susceptible to interobserver variability [3]. Moreover, conventional imaging techniques frequently fail to accurately depict the morphology and distribution of lesions, leading to delays in diagnosis and timely medical intervention. These challenges underscore the necessity for standardized, advanced methodologies to enhance disease management and mitigate public health risks [4]. Recent advancements in deep learning and artificial intelligence have significantly improved medical imaging, particularly in the automated diagnosis of dermatological diseases [5]. Traditional approaches, primarily based on Convolutional Neural Networks (CNNs), rely on localized feature extraction using fixed receptive fields. While CNNs excel at capturing hierarchical visual features, they struggle to model long-range dependencies and global contextual relationships [6]. This limitation becomes especially problematic in analyzing complex lesion patterns, such as those observed in monkeypox, where irregular shapes and heterogeneous textures challenge conventional architectures. In order to overcome these challenges, Transformer-based models have emerged as a powerful alternative. Unlike CNNs, they utilize self-attention mechanisms that enable them to capture both local and global dependencies across an entire image [7]. This ability allows for a more comprehensive representation of lesion morphology, improving the classification of visually similar but morphologically distinct skin abnormalities. By leveraging self-attention, Transformers enhance feature extraction, leading to more accurate and reliable diagnostic outcomes [8].

In addition to improving model performance, integrating eXplainable Artificial Intelligence (XAI) techniques is essential for enhancing interpretability in medical AI applications [9]. These methods provide visual explanations by highlighting the most relevant regions in an image that contribute to a model’s decision [10]. However, visual heatmaps alone might be insufficient for clinical validation. To ensure greater reliability, it is advantageous to complement XAI methods with quantitative metrics that provide numerical confidence scores for interpretability. Techniques such as faithfulness evaluation, region perturbation analysis, and feature importance scoring can offer a more robust assessment of a model’s reasoning, making AI-driven decisions more transparent and trustworthy in clinical settings [11]. By combining Transformer-based architectures with advanced XAI methodologies that quantify interpretability confidence, AI-driven dermatological diagnostics can achieve higher accuracy and greater reliability. These advancements help bridge the gap between deep learning models and real-world clinical applications, fostering trust in AI-assisted medical decision-making.

### 1.1. Research Problem and Significance

Monkeypox, a viral infection characterized by skin manifestations, presents diagnostic challenges due to its visual similarity to other dermatological conditions, such as measles and chickenpox. Traditional deep learning models, particularly CNN-based approaches, have been widely used in medical imaging but often struggle to capture the complex morphological variations in skin lesions. Transformer-based models, such as Vision Transformer (ViT) and Data-Efficient Image Transformer (DeiT), offer advantages in modeling long-range dependencies, yet their decision-making processes remain difficult to interpret due to their self-attention mechanisms [12]. Unlike CNNs, where feature extraction is localized and hierarchical, Transformers distribute attention globally across the image, requiring specialized explainability techniques to understand their predictions [13]. In order to address this interpretability gap, we fine-tuned ViT and DeiT (pre-trained on ImageNet-1K [14]) for classifying monkeypox lesions while distinguishing them from visually similar conditions in a way of more reliable differential diagnosis.

Beyond merely model performance, we emphasize explainability by integrating multiple XAI techniques, including Gradient-weighted Class Activation Mapping (Grad-CAM), Layer-wise Relevance Propagation (LRP), and Attention Rollout (AR). To further enhance interpretability, we generate hybrid XAI heatmaps by applying Principal Component Analysis (PCA) on pairwise combinations of these heatmaps, ensuring more comprehensive visual explanations. Unlike conventional studies where heatmaps are used solely for visualization, we quantitatively validate the generated explanations using causal metrics such as deletion and insertion analysis, providing numerical confidence in the model’s decision-making process.

While CNN-based XAI applications are prevalent in medical imaging, Transformer-based models lack similar interpretability frameworks, limiting their clinical adoption. By systematically implementing and validating explainability methods for ViT and DeiT, this study bridges the gap in understanding how Transformers distinguish monkeypox from other skin diseases, contributing to more transparent and trustworthy AI-driven diagnostics.

This study was conducted in response to the pressing need for reliable and transparent diagnostic tools for monkeypox, particularly given its clinical resemblance to other dermatological conditions and the limitations of existing deep learning methods. While CNN-based models have been widely applied, their restricted receptive fields hinder the analysis of complex lesion structures, and current Transformer models, despite their strengths, remain largely underexplored in this context. Therefore, we propose the use of Transformer-based architectures, specifically ViT and DeiT, as they are well-suited to capture both local and global dependencies in medical images, thereby addressing the unique morphological complexity of monkeypox lesions.

The novelty of this work lies in the integration of multiple Transformer-based classifiers with advanced XAI techniques to provide not only high diagnostic accuracy but also enhanced interpretability. Unlike previous studies that rely primarily on conventional CNN models or apply XAI methods only for visualization, our approach introduces hybrid explanation maps and quantitative causal metrics to validate interpretability. By doing so, this study contributes to the literature by bridging the gap between performance and explainability in Transformer-based monkeypox classification, offering a clinically meaningful framework that enhances trust and usability in real-world healthcare applications.

### 1.2. Related Work

The literature is categorized into two main groups: studies that incorporate XAI and those that do not. It has been observed that the Monkeypox Skin Lesion Dataset (MSLD) and the Monkeypox Skin Images Dataset (MSID), which includes images from MSLD, are frequently used. In addition, the 6-class Monkeypox Image Dataset (MID) includes the subsets used for both the 2-class and 4-class tasks. A small number of studies have also constructed their own image datasets using open-source data.

#### 1.2.1. Studies Utilizing XAI Methods

In recent years, XAI methods have been increasingly used to enhance the interpretability of deep learning models in medical image analysis. In the context of monkeypox detection, studies have primarily employed techniques such as Local Interpretable Model-agnostic Explanations (LIME) and Grad-CAM to highlight relevant image regions and improve model transparency. In this section, we review studies that integrate these XAI approaches, analyzing their impact on model interpretability and diagnostic reliability.

Lakshmi and Das utilized a pre-trained ResNet101 model and achieved the highest accuracy of 94.25% on the MSLD. Furthermore, they incorporated LIME to enhance the interpretability of their model [15]. Ahsan et al. modified and tested six pre-trained models using transfer learning. Their results showed that InceptionResNetV2 and MobileNetV2 models achieved the highest accuracy, ranging from 93% to 99%. Additionally, they applied LIME to enhance the model’s interpretability by identifying key features related to monkeypox. In their study, they utilized two versions (augmented, non-augmented) of a 2-class dataset consisting of monkeypox and non-monkeypox cases, one with data augmentation and one without, reaching different results for each approach [16]. Raha et al. proposed an attention-based MobileNetV2 model for monkeypox detection. The model’s interpretability was supported using Grad-CAM and LIME. In their study, they utilized two different datasets and achieved the best performance, with an accuracy of 98.19%, on the MSID [17]. Saavedra et al. achieved an accuracy of 98% using an ensemble of ResNet50, EfficientNetB0, and MobileNetV2 models. Moreover, this study is the first to apply XAI techniques, such as Grad-CAM, with ensemble networks. In their study, a 3-class monkeypox skin dataset was used for evaluation [18]. Bala et al. proposed a modified DenseNet-201-based CNN model, called MonkeyNet, which achieved 93.19% accuracy on the MSID and 98.91% with an augmented dataset. They visualized infected regions within Grad-CAMs [19]. Azar et al. proposed a DenseNet201 model-based architecture in their study, achieving an accuracy of 97.63% on MSLD and an accuracy of 95.18% on MSID. Furthermore, they applied Grad-CAM and LIME techniques to interpret the provided results [20]. Sitaula and Sihahi proposed a comparison of 13 different pre-trained deep learning models. After identifying the best-performing models, they ensembled them using a majority voting strategy over the probabilistic outputs. Their approach, evaluated on MSID, achieved an average accuracy of 87.13%. As well, they applied Grad-CAM and LIME techniques to enhance model interpretability [21]. Nayak et al. proposed a method that involved training and evaluating five pre-trained deep neural networks. They achieved the highest accuracy of 99.49% with the ResNet-18 model, while all modified models surpassed 95% validation accuracy. Furthermore, LIME and Grad-CAM were proposed to enhance model interpretability in diagnosing monkeypox. Their study utilized the augmented MSLD for evaluation [22]. Aloraini proposed a method that fine-tunes a ViT model. In addition, Grad-CAM was used as an explanation method and achieved an accuracy of 94.6% using an augmented MSLD [23]. Siddick et al. demonstrated that the ViT and hybrid model outperformed other models in terms of testing accuracy, showing strong potential for accurate and interpretable monkeypox diagnosis. ViT obtained 99.41% and the hybrid model achieved 99.09% testing accuracy. The ViT and hybrid models, supported by XAI techniques like Grad-CAM, enhanced prediction visualization. Their study utilized MID for evaluation [24]. Setegne et al. used a GitHub dataset of 211 monkeypox cases with 47 clinical symptoms, expanded to 372 samples via SMOTE to balance classes. Ensemble ML models (Random Forest, Bagging, Gradient Boosting, CatBoost, XGBoost, LGBMClassifier) with XAI techniques (SHAP, LIME, ELI5, counterfactuals, Qlattice) were applied for symptom-based monkeypox detection, with Featurewiz for feature selection. The LGBM Classifier achieved 89.3% accuracy [25]. Kumar Saha et al. used the MSID with 770 images across four classes, resized to 224 × 224. The proposed monkeypox-XDE ensemble combined modified Xception, DenseNet201, and EfficientNetB7 models via Softmax, dense, flattened layers, and 65% dropout, with Swin Transformer (SwinViT) for comparison. Feature extraction and Grad-CAM XAI were applied for interpretability. Monkeypox-XDE achieved 98.70% accuracy, 98.90% precision, 98.80% recall, and 98.80% F1-score on testing data [26]. Nasiri et al. used the MSLD dataset. The proposed method combined Xception for feature extraction, PCA for dimensionality reduction, NGBoost for classification, and African Vultures Optimization Algorithm (AVOA) for hyper-parameter tuning, with Grad-CAM and LIME for explainability. It achieved 97.53% accuracy, 97.72% F1-score, and 97.47% AUC, demonstrating high performance and interpretability for monkeypox detection in resource-constrained settings [27].

#### 1.2.2. Studies Without XAI Methods

While many studies have incorporated XAI techniques to enhance interpretability, a significant number of works on monkeypox detection rely solely on deep learning models without explainability methods. These studies primarily focus on classification performance, often evaluating models based on accuracy, precision, and recall, without providing insights into the decision-making process. Most of these approaches employ CNN and Transformer architectures to extract features and classify images, yet they do not include additional mechanisms to interpret the model’s predictions. In this section, we review studies that have adopted deep learning models without XAI.

Kumar employed three different CNN architectures. The results of the study demonstrated that the highest accuracy of 91.11% was achieved when utilizing VGG16 model-derived features with the Naive Bayes classifier. Their study was conducted using the MSLD [28]. Altun et al. developed a hybrid CNN model. The study tested several models. The optimized hybrid MobileNetV3-s model performed the best, achieving an average accuracy of 96%. In their study, they created their own dataset by utilizing open-source monkeypox data [29]. Uysal used state-of-the-art deep learning models for monkeypox detection using MSID. To enhance classification results, they proposed a hybrid model that combined the two best-performing models with a long short-term memory network. The developed system achieved a test accuracy of 87% and a Cohen’s kappa score of 0.8222 [30]. Abdelrahim et al. presented an ensemble model that combines Transformer models and SVM, along with an evaluation of seven CNN architectures. The four top-performing models were selected for feature extraction, and the high-dimensional feature vectors extracted from these models were then combined, optimized, and passed into an SVM classifier for final classification. They evaluated their approach using a 2-class augmented dataset, achieving the highest accuracy of 95.45%, demonstrating the model’s robustness to variations in the data [31]. Kundu et al. proposed a method that incorporates a cycle-consistent generative adversarial network for data augmentation and classification, along with a flower federated learning system to ensure data security. Their experimental results demonstrated the effectiveness of the proposed approach, with the ViT-B32 model achieving an accuracy of 97.90% [32]. Oztel proposed a system for classifying seven skin lesions, including monkeypox [33]. In this approach, a ViT and several popular deep learning convolutional networks were trained using transfer learning. The proposed ensemble-based system achieved 81.91% accuracy. They used a combined dataset (MSLD and PAD-UFES-20). Haque et al. implemented five deep learning models incorporating both channel and spatial attention mechanisms. A comparative analysis revealed that the Xception-CBAM-Dense architecture outperformed the other models, achieving a validation accuracy of 83.89% in classifying monkeypox and other diseases [34]. Srinivasan et al. utilized a custom dataset of 659 skin images with data augmentation. The proposed ESACN (Enhanced Spatial-Awareness Capsule Network) model, an advanced CapsNet with a spatial-awareness mechanism, achieved 92.68% accuracy, 78.90% precision, 88.64% recall, and 82.04% F1-score for multi-class classification, demonstrating robustness on limited data [35]. Cao et al. utilized the MSLD dataset, ISIC 2018 (seven skin diseases), Dermnet (eight skin diseases), and a clinical dataset (146 patient images + 40 normal skin). The proposed “Mask, Inpainting, and Measure” (MIM) framework employed GAN for inpainting masked monkeypox images, treating monkeypox as normal and non-monkeypox as anomalous, with SSIM and histogram Bhattacharyya distance for scoring. It achieved 0.8237 AUROC (average), outperforming ResNet50 (0.6221), MobileNetV3 (0.5755), and Swin Transformer (0.6306), with a clinical AUROC of 0.871 [36]. Maheskumar et al. utilized the MSLD with 3192 augmented images. The proposed ResViT-FLBoost framework integrated federated learning with hybrid ResNet-ViT, adaptive attention, and XGBoost-LightGBM ensemble classifiers, using adaptive thresholding and CLAHE for preprocessing. It achieved 98.78% accuracy, with superior precision, recall, and robustness, enabling privacy-preserving, scalable monkeypox detection in decentralized healthcare settings [37]. Pal et al. used the Monkeypox Skin Lesion Dataset v2.0 with six lesion categories. The ViT model outperformed traditional ML models, achieving 93.03% accuracy in detecting Mpox lesions. An ensemble combining ViT and ConvMixer further improved diagnostic performance, reaching 94% accuracy, 94% precision, 97% recall, 96% F1-score, and 98% AUC for Mpox classification [38].

In the literature, CNN-based approaches have been predominantly proposed, with a limited number of studies incorporating Transformer-based and hybrid models. XAI-based studies have primarily utilized methods such as Grad-CAM and LIME. In the proposed study, these gaps are addressed by employing Transformer-based classifiers and exploring innovative XAI methods beyond conventional techniques to evaluate their effectiveness.

## 2. Materials and Methods

**Figure 1:**
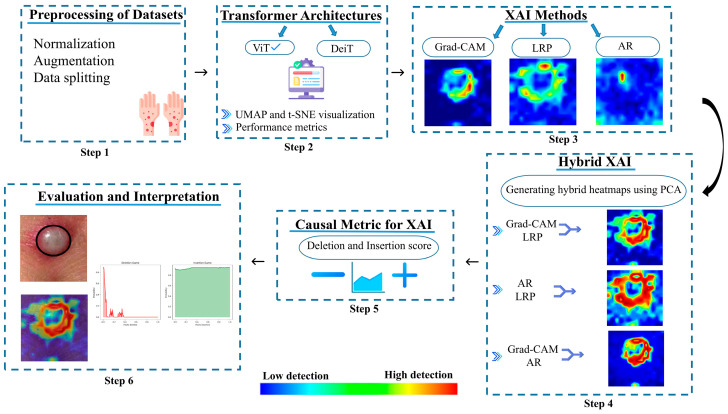
Overview of the methodological workflow.

The image classification pipeline utilizing MSLD and MSID via Transformer architectures can be seen in Figure 1. The preprocessing of current datasets may be observed at the initial stage (Step 1). Furthermore, two well-known Transformer architectures, namely ViT and DeiT, are utilized as deep neural architectures. Well-curated data preparation is the first step in the training and testing process. Both ViT and DeiT are fine-tuned from pre-trained Imagenet-1k weights. Feature distributions of Transformer architectures are visualized within Uniform Manifold Approximation and Projection (UMAP) and t-Distributed Stochastic Neighbor Embedding (t-SNE). Finally, performance metrics of test sets are reported. In the third step, Grad-Cam, LRP and AR heatmaps are generated for each image in the testing set with an explainable approach. We propose a PCA-based novel hybrid image generation approach to come up with more effective explainable images, which we perform in Step 4. Then, we evaluate how these generated heatmaps highlight the regions of interest (ROIs) across all classes using causal evaluation metrics. Finally, we put forward a comparative analysis to evaluate which Transformer architecture performs well within an effectively selected explainable method for the image classification problem in MSLD and MSID.

### 2.1. Datasets

In this study, the MSLD and MSID, widely used and significant datasets in the literature, are selected [19,39]. The MSLD contains skin images and lesions specific to monkeypox, providing researchers with a comprehensive resource for developing, training, and testing various machine learning and deep learning models. This dataset is divided into two classes: Monkeypox (102 images) and Others (126 images, including measles and chickenpox), totaling 226 images with a relatively balanced distribution. Although the dataset includes folders with augmented images, only the original images are used in this study, with augmentation applied dynamically during the training phase. Moreover, this study also incorporates the MSID, which includes four distinct classes: Monkeypox (279 images), Chickenpox (107 images), Measles (91 images), and Normal (293 images). To be more specific, as mentioned earlier, monkeypox is a zoonotic viral infection characterized by fever, headache, muscle aches, and a rash resembling smallpox. It is primarily transmitted through contact with infected animals or humans and can lead to severe complications, especially in immunocompromised individuals [40]. Chickenpox is caused by the varicella-zoster virus, and presents as an itchy, blister-like skin rash. While often mild in children, it can lead to serious complications such as pneumonia and encephalitis, particularly in adults and immunocompromised individuals [41]. Measles is a highly contagious viral disease marked by symptoms including high fever, cough, runny nose, red eyes, and a characteristic red blotchy skin rash. Measles can lead to severe complications like pneumonia and encephalitis, especially in young children and adults [42]. The Normal class represents healthy skin tissue without any lesions or abnormalities, serving as a baseline for comparison in diagnostic imaging.

### 2.2. Proposed Methodology

#### 2.2.1. Data Preparation

Due to the limited number of images in the datasets, several techniques are employed to mitigate overfitting. The datasets are split into 80% training and 20% testing, with 10% of the training data further allocated for validation. The images are resized to 224 × 224 pixels. Moreover, image normalization is applied to standardize pixel values across the dataset. The mean ([0.485, 0.456, 0.406]) and standard deviation ([0.229, 0.224, 0.225]) values, derived from the ImageNet dataset, are used to ensure consistency with pre-trained models. This process adjusts each pixel by subtracting the mean and dividing by the standard deviation, improving model stability and convergence during training.

#### 2.2.2. Transformer Architecture

(a) Vision Transformer (ViT)

Traditional CNNs have been widely used for image classification tasks due to their ability to capture spatial hierarchies in images through convolutional operations. However, these architectures inherently suffer from limitations in capturing long-range dependencies due to their local receptive fields. To overcome these challenges, ViT, introduced by Dosovitskiy et al. [43], replaces convolutional operations with a self-attention mechanism, allowing global context modeling across an image. ViT achieves this by treating an image as a sequence of non-overlapping patches and processing them using Transformer-based architectures originally designed for natural language processing.

The ViT model follows the standard Transformer encoder structure proposed by Vaswani et al. [44]. Instead of processing raw image pixels through convolutions, ViT splits an image into non-overlapping patches of fixed size. These patches are then flattened and passed through a linear projection layer to obtain patch embeddings. Formally, $x\epsilon R^{H\times W\times C}$, given an input image, where H and W are height and width, and C is the number of channels. The image is divided into N patches of size P×P, resulting in $N=\frac{HW}{P^{2}}$ total patches. Each patch is projected into a one-dimensional embedding space, forming a sequence of embeddings as given in Equation (1).

(1) $$Z_{0}={[X}_{1\;}E;\;X_{2\;}E;\ldots ;X_{n}E]+E_{pos}$$

Here, E refers to the learnable embedding matrix. $E_{pos}$ represents the positional encodings, which help the Transformer retain spatial information. Since Transformers are permutation-invariant, they do not inherently preserve positional relationships. The addition of positional encodings ensures that the model can capture the spatial structure of the input data.

**Figure 2:**
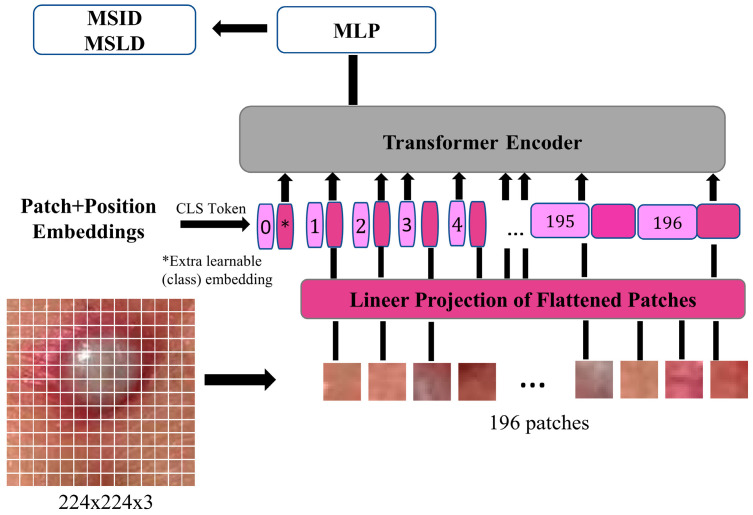
ViT model representation of monkeypox image.

The Transformer encoder consists of L layers, each incorporating key components to ensure efficient learning and representation. The Multi-Head Self-Attention (MSA) mechanism captures dependencies across different image patches, allowing the model to establish global contextual relationships. Following this, the Feedforward Network, which is a conventional multi-layer perceptron, applies a two-layer fully connected network with Gaussian Error Linear Unit (GELU) activation [45], enhancing the model’s capacity to learn complex feature representations. To ensure training stability, layer normalization is applied. Additionally, residual connections help maintain a smooth gradient flow, which prevents vanishing gradients. This mechanism also enhances convergence, leading to more efficient training. The architecture of the ViT model used in this study is presented in Figure 2.

Each Transformer encoder layer processes the sequence of embeddings using self-attention, as shown in Equation (2):

(2) $$Attention(Q,K,V)=\;\mathrm{softmax}\left(\frac{{QK}^{T}}{\sqrt{d_{k}}}\right)V,$$

where queries (Q), keys (K), and values (V) are linearly projected representations of the input embeddings, and $d_{k}$ is the scaling factor. At the final stage, a classification token [CLS] is appended to the input sequence, and its final embedding is used for classification through a fully connected multi-layer perceptron (MLP) head [46].

(b) Data Efficient Vision Transformer (DeiT)

DeiT, introduced by Facebook AI, builds upon the ViT architecture but is designed to improve training efficiency, particularly for smaller datasets and more resource-constrained environments [47]. While the ViT model requires large amounts of data and computational resources for effective training, DeiT employs a knowledge distillation approach, which leverages a larger teacher model to train a smaller student model. This method accelerates convergence and improves generalization capabilities, making DeiT more practical for a wide range of image classification tasks, especially when training data is limited.

The DeiT architecture closely follows the ViT model, utilizing a self-attention mechanism to model global dependencies across image patches. However, DeiT enhances this approach by incorporating a Feedforward Network (FFN) above the MSA layer. The FFN consists of two linear layers, separated by a GELU activation, which allows the model to capture more complex feature representations. Furthermore, DeiT’s structure includes positional encodings to preserve spatial information, ensuring the model can capture the relative positions of patches despite the permutation-invariant nature of Transformers.

#### 2.2.3. Fine-Tuning Transformer-Based Architectures

The augmentation techniques employed include random horizontal flip, random rotation, color jitter, random resized crop, random vertical flip, random affine, random perspective, and Gaussian blur. These transformations were selected based on commonly used methods in the literature known to improve model robustness and generalization capability by enabling the model to handle real-world data variations more effectively. To better illustrate the applied augmentations, Table 1 presents an original monkeypox lesion image alongside its transformed versions. For example, horizontal and vertical flips simulate orientation variability, random rotations and affine transformations mimic camera angle changes, and color jitter accounts for lighting variations. Perspective transformations and Gaussian blur emulate realistic distortions or imaging noise that may occur in real-world clinical settings.

In addition to these augmentations, regularization and optimization techniques such as cut–mix [48], mix-up [49], label smoothing [50], and early stopping were utilized to prevent overfitting. First, CutMix and MixUp were applied randomly to combine training images and their labels, encouraging the model to generalize better by reducing reliance on specific features of individual samples. Label smoothing with a factor of 0.1 was used to prevent the model from becoming overconfident on the training labels. We also implemented early stopping, halting training if the validation loss did not improve for 15 consecutive epochs. Finally, a small learning rate of 0.0001 was adopted to ensure stable convergence and reduce the risk of overfitting. These combined techniques helped improve the model’s generalization performance on unseen data.

The specific hyper-parameters used for these methods are detailed in Table 2. The selection of these hyper-parameters was guided by a combination of literature defaults and pilot experiments involving limited grid search and validation sweeps on a subset of the training data. Alternative values were tested during preliminary experiments, and the ones reported in Table 2 demonstrated the best performance in terms of validation accuracy and loss stability. Furthermore, the same hyper-parameter settings were applied consistently across both the 2-class and 4-class classification tasks to ensure a fair comparison between models. Preliminary tuning indicated that these parameters yielded near-optimal results for both tasks, and thus, separate tuning for each task was not performed.

Each model was initialized with pre-trained weights and fine-tuned by replacing the classification head with a fully connected layer tailored to the specific classification task. Optimized model weights were saved upon completion of training for further analysis and testing.

#### 2.2.4. Visual Exploration of Feature Embeddings

t-SNE and UMAP are widely used dimensionality reduction techniques for visualizing high-dimensional data. t-SNE preserves local similarities between data points by minimizing the Kullback–Leibler divergence between high-dimensional and low-dimensional probability distributions, making it effective for capturing complex nonlinear structures [51]. In contrast, UMAP is a graph-based technique that leverages topological structure preservation and geometric assumptions to achieve faster and more efficient dimensionality reduction, particularly for large datasets [52].

In this study, ViT and DeiT models are fine-tuned for the binary and multi-class classification problem. After training, feature representations are extracted from the penultimate layer of each model by removing the classification head. These extracted features are then projected into a two-dimensional space using t-SNE and UMAP to analyze the discrimination of the learned feature embeddings. The visualizations provide insight into how well the models differentiate between the 2 classes and 4 classes in the feature space, with distinct clusters indicating a well-generalized representation. By leveraging these dimensionality reduction techniques, the study aims to interpret the learned feature representations and assess the effectiveness of Transformer-based architectures in distinguishing between classes. The results demonstrate the clustering behavior of the feature embeddings, further contributing to the explainability of the classification models.

#### 2.2.5. XAI-Based Methods

XAI is a research field focused on improving the transparency and interpretability of AI models. It addresses the challenges associated with traditional black-box systems. As machine learning models, particularly deep learning architectures, are increasingly used to make critical decisions, understanding their inner workings is crucial for trust, accountability, and ethical deployment. The goal of XAI techniques is to provide human-interpretable explanations for model predictions. This enables users to understand how and why specific decisions are made. This is especially important in high-stakes domains, such as healthcare, where the reliability and explainability of AI-driven decisions directly influence patient safety and clinical outcomes. By bridging the gap between model performance and interpretability, XAI supports informed decision-making, regulatory compliance, and the broader adoption of AI in sensitive applications [53].

In the current study, three different XAI methods, namely Grad-CAM, Rollout, and LRP, are utilized. Moreover, their parwised hybrids are innovatively generated using PCA to improve heatmap explanations.

(a) Grad-CAM

In this section, we first describe the Grad-CAM technique, a gradient-based localization method originally developed for CNNs [54]. Grad-CAM leverages the gradients of the target class score with respect to the activations in a convolutional layer to produce a coarse localization map that highlights image regions contributing positively to the prediction. Formally, let $y^{c}$ denote the pre-softmax score for class *c* and $A^{k}$ the $k_{th}$ feature map obtained from the last convolutional layer. The neuron importance weights $\alpha_{k}^{c}$ are computed by globally averaging the gradients over the spatial dimensions as given in Equation (3):

(3) $$\alpha_{k}^{c}=\frac{1}{Z}\sum_{i}\sum_{j}\frac{\partial y^{c}}{{\partial A}_{ij}^{k}},$$

where *Z* is the total number of spatial locations. The final class-discriminative localization map is then obtained via a weighted combination of the activation maps, followed by a *ReLU* operation to retain only the features with a positive influence on $y^{c}$ as given below in Equation (4).

(4) $$L_{Grad-CAM}^{c}=ReLU\left(\sum_{k}\alpha_{k}^{c}A^{k}\right)$$

Next, we extend this approach to ViTs. Unlike CNNs, ViTs process images by dividing them into non-overlapping patches that are subsequently embedded into a sequence, augmented with positional encodings, and processed via self-attention mechanisms [55]. Given a target class *c*, the gradient of the pre-softmax score $y^{c}$ is computed with respect to the patch embeddings $Z_{i}$, where each patch embedding corresponds to a token in the Transformer architecture. The importance weight for each patch is determined by averaging the gradients over the embedding dimensions in Equation (5):

(5) $$\alpha_{i}^{c}=\frac{1}{D}\sum_{j=1}\frac{{\partial y}^{c}}{{\partial Z}_{i,j}},$$

where *D* is the total number of embedding dimensions. The class-discriminative localization map is then obtained by computing a weighted combination of the patch representations and applying a *ReLU* activation to retain only the contributions that positively influence $y^{c}$ in the following formula:

(6) $$L_{ViT-GradCAM}^{c}=ReLU\left(\alpha_{1}^{c},\alpha_{2,}^{c}\ldots \alpha_{N}^{c}\right)$$

Since ViTs do not generate spatial feature maps like CNNs, the resulting importance scores are bilinearly interpolated to align with the original image dimensions as reported in [56], and given by Equation (7).

(7) $$L_{ViT-GradCAM}^{c}=Interp\left({Reshape(L}_{ViT-GradCAM}^{c},\surd Nx\surd N),H,W\right)$$

This Transformer Grad-CAM not only preserves the interpretability benefits of the original Grad-CAM by highlighting class-relevant regions but also provides insights into the self-attention mechanisms that drive the model’s predictions. By leveraging the inherent patch-based structure of ViTs, this method enables a fine-grained visualization of how individual patches contribute to the classification outcome.

(b) LRP

LRP is a gradient-based interpretability method designed to explain the decision-making process of deep learning models by propagating relevance scores from the output layer back to the input features [57]. The main idea of LRP is to decompose the model’s output prediction f(x) for a given input *x* into contributions from individual input features, ensuring that the sum of these contributions equals the output score given by Equation (8):

(8) $$f\left(x\right)=\sum_{i}R_{i},$$

where $R_{i}$ denotes the relevance score of the input feature. In the context of ViTs, LRP is particularly effective in analyzing the self-attention mechanisms, which capture global dependencies between image patches. Given a target class *c*, the relevance propagation starts from the output logit $y^{c}$ and distributes it layer-by-layer through the network using a set of conservation rules. For a linear layer with weights w and activations a, the relevance $R_{j}$ of neuron j in the previous layer is computed as in Equation (9):

(9) $$R_{j}=\sum_{k}\frac{a_{j}W_{jk}}{\sum j^{\prime }a_{j^{\prime }}W_{j^{\prime }k}}R_{k},$$

where $R_{k}\;$is the relevance of neuron k in the current layer. This ensures that total relevance is conserved across layers.

In ViTs, LRP is adapted to analyze the self-attention mechanisms, which capture global dependencies between image patches. For the last-layer LRP approach, the relevance scores are computed using the attention maps (A) and gradients (G) from the final self-attention layer. The relevance R for each patch is derived as in Equation (10):

(10) $$R=\frac{1}{H}\sum_{h=1}^{H}max(G_{h}⊙A_{h},0),$$

where H is the number of attention heads, $G_{h}$ and $A_{h}$ are the gradient and attention map for the h-th head, respectively, and ⊙ denotes element-wise multiplication. The max (⋅, 0) operation ensures that only positive contributions are retained. Finally, the relevance scores are averaged across heads, and the CLS token is excluded to focus on image patches as $R_{patches}=R\left[1\;:\right].$ This method generates a class-discriminative heatmap, highlighting the image regions most influential to the model’s prediction [55].

(c) AR

AR is a method for quantifying information propagation across layers in Transformer models, providing insights into how attention mechanisms distribute input information throughout the network [58]. Given a Transformer with L layers, Attention Rollout recursively accumulates attention weights to compute the total contribution of input tokens to deeper layers. For a given layer $l_{i}$ and an earlier layer $l_{j}$ where j<i, the cumulative Attention Rollout $Ã\left(l_{i}\right)$ is defined as follows:

(11) $$Ã\left(l_{i}\right)=\left\{\begin{array}{c} A\left(l_{i}\right)Ã\left(l_{i-1}\right),\;if\;i>j\\ A\left(l_{i}\right),\;if\;i=j \end{array}\right\},$$

where A(l) represents the raw attention matrix and Ã(l) denotes the accumulated Attention Rollout. This formulation ensures that attention weights are propagated backward across all layers, allowing for a structured analysis of information flow. By setting j=0, it is possible to compute input attention, revealing the influence of each input token on the model’s final representation.

In this study, AR is employed to generate class-specific visual explanations by aggregating attention contributions across all layers. This method enables a fine-grained visualization of how information is transmitted from the input layer to the final decision, offering deeper interpretability of the Transformer’s decision-making process. By leveraging Attention Rollout, it becomes possible to highlight the regions of an input image that most significantly contribute to a classification outcome, thus improving model transparency and trustworthiness.

(d) Hybrid explainable heatmap generation using PCA

In this study, PCA is employed to merge heatmaps generated by two different XAI methods. PCA is a dimensionality reduction technique that identifies the directions capturing the most variance in high-dimensional data [59]. First, since some of the highlighted pixels in the Grad-CAM heatmaps are located outside the lesion region, while the actual lesion areas are represented with lower intensity, a histogram analysis is applied to the Grad-CAM outputs. This step helps suppress irrelevant boundary regions by assigning them to the background. To further highlight the lesion and eliminate unrelated areas, the Connected Components function from the OpenCV library is used. Finally, the intensity values of both heatmaps are combined at the pixel level, forming a two-dimensional feature vector in Equation (12).

(12) $$x_{i}=\left[\begin{array}{c} h_{1,i}\\ h_{2,i} \end{array}\right]$$

In order to ensure that the principal components reflect variance rather than being influenced by central tendencies, the mean intensity is computed and subtracted as follows:

(13) $$\mu =\frac{1}{N}\sum_{i=1}^{N}x_{i}$$

A covariance matrix is then constructed to capture the relationships between the two heatmaps as given below:

(14) $$C=\frac{1}{N}\sum_{i=1}^{N}{\left(x_{i}-\mu )(x_{i}-\mu \right)}^{T},$$

which is followed by eigen decomposition to extract the principal components as Cv=λ*v*. The eigen vector corresponding to the largest eigen value is selected as the first principal component, representing the direction of maximum variance. This principal component is used to project the two-dimensional data into a single dimension as $y_{i}=v_{1}^{T}(x_{i}-\mu )$, yielding a hybrid heatmap that effectively fuses the most informative aspects of both original heatmaps. Finally, a min–max normalization is applied to scale the heatmap values between 0 and 1, ensuring consistency in visualization and comparability across different datasets. This approach integrates the explanatory information from both XAI methods, producing a more interpretable and comprehensive representation.

#### 2.2.6. Causal Metrics with XAI

Explainability in machine learning has become a critical area of research as models grow increasingly complex. The ability to interpret and understand the decisions made by these models is essential for ensuring transparency, trust, and accountability. To evaluate the effectiveness of XAI methods, researchers have proposed a variety of metrics that quantify different aspects of explainability. These metrics can be broadly categorized into faithfulness, robustness, and complexity measures [60,61].

Faithfulness metrics assess how well the explanations align with the actual reasoning process of the model. For instance, deletion and insertion scores are widely used to evaluate the importance of features identified by an XAI method. The deletion metric measures the drop in model performance when the most important features, as identified by the explanation, are removed. Conversely, the insertion metric evaluates the improvement in model performance when these features are gradually reintroduced [62]. These metrics are computed using the causal metric framework, which evaluates the change in model confidence as pixels are progressively deleted or inserted based on the importance scores provided by the heatmaps.

In this study, the deletion metric is used to assess how much the model’s predictions rely on the most important areas of an image. First, the images are resized to 224 × 224, and heatmaps of the same size are generated. These heatmaps, normalized to a [0, 1] range, indicate the importance of each pixel according to the model. The model’s prediction function f(.) takes an image as input and outputs the softmax probabilities. The deletion metric works by gradually removing the most important pixels, as identified by the heatmap, and measuring the resulting decrease in the model’s confidence.

Let $S_{d}$ be the set of pixels ranked by their importance scores in descending order. The deletion metric D(t) measures the drop in model confidence when the top t% of pixels are removed, defined as follows:

(15) $$D\left(t\right)=f(Ῑ⊙M_{d}\left(t\right)),$$

where Ῑ is the normalized input image, ⊙ denotes element-wise multiplication, and $M_{d}(t)$ is a binary mask that removes the top t% of pixels. At each step, the modified image $Ῑ⊙M_{d}\left(t\right)$ is processed by the model, and the confidence score $f(Ῑ⊙M_{d}\left(t\right))$ is computed. This process creates a curve that illustrates how the model’s confidence declines as the most important pixels are gradually removed. The deletion metric measures the model’s reliance on these key regions by tracking the drop in prediction confidence. The area under the curve (AUC) quantifies this dependence, offering insight into how crucial these regions are for the model’s predictions. A steep decline in the curve suggests a strong reliance on the removed pixels, emphasizing their significance in the model’s decision-making process.

On the other hand, the insertion metric is employed to evaluate how the model’s confidence increases as the most important pixels are gradually reintroduced into the image. The preprocessing steps for the insertion metric closely follow those of the deletion metric. The insertion metric is based on the principle of iteratively reintroducing the most important pixels, as identified by the heatmap, and measuring the resulting increase in the model’s confidence. Let $S_{i}$ be the set of pixels ranked by their importance scores in ascending order. The insertion metric I(t) measures the increase in model confidence when the top t% of pixels are gradually reintroduced, defined as given below:

(16) $$I\left(t\right)=f(Ῑ⊙M_{i}\left(t\right)),$$

where $M_{i}(t)$ is a binary mask that inserts the top t% of pixels. To simulate the gradual reintroduction of information, a Gaussian blur is applied to the background regions, ensuring a smooth transition. At each step, the modified image $Ῑ⊙M_{i}\left(t\right)$ is processed by the model, and the confidence score $f(Ῑ⊙M_{i}\left(t\right))$ is computed. This process generates a curve that shows how the model’s confidence increases as the most important pixels are progressively reintroduced. The insertion metric assesses the model’s sensitivity to the most important regions by gradually reintroducing them and measuring the increase in prediction confidence. AUC measures the model’s responsiveness to the reintroduction of the most salient regions, demonstrating how effectively the model can recover its confidence as these regions are gradually restored. A steep increase in the curve suggests that the model is highly sensitive to the presence of these critical features.

## 3. Results

### 3.1. Feature Embedding Distributions Extracted by Transformer Models

**Figure 3:**
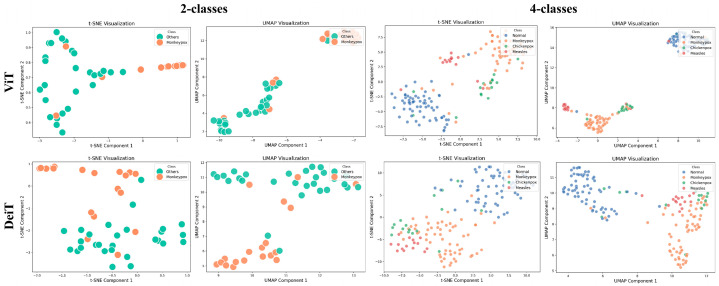
t-SNE and UMAP visualizations of feature embeddings extracted by Transformer models.

In the post-training phase, the quality of the extracted features and clustering performance are thoroughly analyzed using visualization techniques such as t-SNE and UMAP, despite the limited number of images in the dataset. For the MSLD, which contains only two classes (Monkeypox vs. Other), the clusters are clearly separated, reflecting the relative ease of discriminating between the two categories. In contrast, for the MSID, which includes four classes (Monkeypox, Chickenpox, Measles, and Normal), the visualizations show that the Normal class is well-separated from the other three, while Monkeypox, Chickenpox, and Measles exhibit overlapping clusters due to their visual similarities. These observations highlight that more advanced classifiers, such as the ViT model, are necessary to capture subtle distinctions between similar classes. As illustrated in Figure 3, the ViT model produces more distinct and well-separated clusters compared to DeiT, emphasizing its superior ability to encode complex data structures and achieve effective class discrimination. Overall, these results underscore the importance of robust deep learning approaches for Monkeypox diagnosis, particularly in multi-class scenarios where visual similarity poses a challenge.

### 3.2. Classification Results of Test Set Images Utilizing ViT and DeiT

In the current study, ViT and DeiT models are trained over 50 epochs, and their train/validation accuracy and loss graphs, confusion matrices and ROC-AUCs are given in Table 3 and Table 4. Based on the train–validation trends, 50 epochs were sufficient for convergence, as extending training to more epochs resulted in negligible improvements in accuracy and minimal loss reduction. Since we fine-tuned pre-trained networks and applied early stopping with a patience of 15 epochs, longer training was unnecessary and would only increase computational cost without meaningful benefit. Performance metrics of both Transformer methods are given in Table 5 and Table 6. ViT achieves higher accuracy and overall performance with an AUC of 0.9192 for MSLD and 0.9784 for MSID.

According to Table 5, ViT achieves perfect recall (1.0000) for the “Others” class, while maintaining a high precision (0.8750), resulting in a strong F1-score of 0.9333. This indicates that the model is able to correctly identify almost all “Others” samples without a substantial number of false positives. While ViT achieves 100% recall on the “Others” class in the MSLD dataset, several factors indicate that this does not necessarily reflect overfitting. First, we monitored the train–validation accuracy and loss throughout training, which showed stable convergence without signs of overfitting. Second, early stopping with a patience of 15 epochs was applied to prevent the model from memorizing the training data. Third, we report precision, recall, and F1-score for each class; the F1-score balances precision and recall, providing a more reliable measure of the model’s performance than recall alone. The F1-score for the “Others” class (0.9333) confirms that the high recall is accompanied by a good precision (0.8750), suggesting the model is correctly classifying this class without overfitting.

Since the ViT model produced the best results, it is assumed that the heatmaps generated from it would also yield superior outcomes; therefore, all heatmap applications are conducted using ViT. Table 7 and Table 8 present the causal metric results for MSLD and MSID within different XAI methods. In our hybrid XAI approach, only Grad-CAM and LRP were combined to generate paired heatmaps. Rollout was excluded because, as shown in Table 7 and Table 8, it produces higher deletion scores and lower insertion scores compared to the paired hybrid, indicating less reliable localization of essential features. The hybrid approach thus maximizes the preservation of important regions while minimizing irrelevant areas, providing more accurate and interpretable explanations. The paired hybrid approach, derived from Grad-CAM and LRP, achieves the averaged insertion score (0.865 ± 0.005) and deletion score (0.209 ± 0.026) for MSLD and average insertion score (0.899 ± 0.004) and deletion score (0.192 ± 0.010) for MSID, indicating its superior ability to preserve essential features while minimizing irrelevant ones. The Normal class in MSID is not considered due to no lesion indications. These values are computed as the average across all test set images, ensuring that the evaluation of XAI methods is not solely based on visual interpretation but also supported by causal metrics.

**Figure 4:**
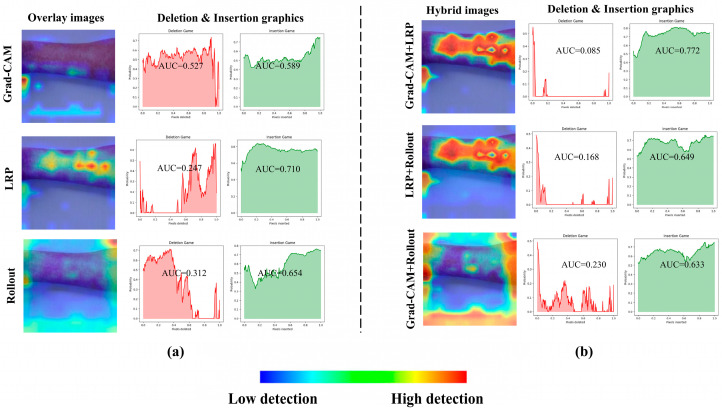
A sample result and deletion–insertion graphs for Grad-CAM, LRP, AR (**a**) and their hybrid approach (**b**).

Figure 4 presents the overlaid results obtained from a sample measles image using Grad-CAM, LRP, and AR explainability methods, along with their corresponding deletion and insertion graphs (see Figure 4a). These visualizations highlight the distinct regions of interest identified by each method, providing insights into how the model prioritizes different features during decision-making. As evident from these results, the hybrid version of Grad-CAM and LRP, achieved through PCA, demonstrates the best performance, offering sharper and more interpretable heatmaps that align closely with the ground truth (see Figure 4b). This superiority is further supported by the quantitative graphs, where the deletion curve for the hybrid method shows a rapid and steep decline, indicating that the model loses its predictive power almost immediately when critical regions are removed. This behavior contrasts with the more gradual decline observed in Grad-CAM and LRP individually, underscoring the hybrid approach’s ability to focus intensely on the most relevant features. The insertion metrics also confirm the hybrid method’s effectiveness, as it achieves higher precision in restoring model performance when key regions are progressively added, solidifying its robustness in capturing discriminative features for accurate classification.

**Figure 5:**
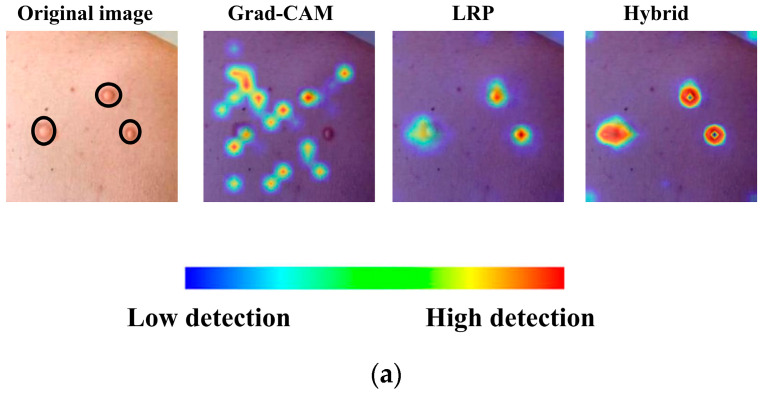

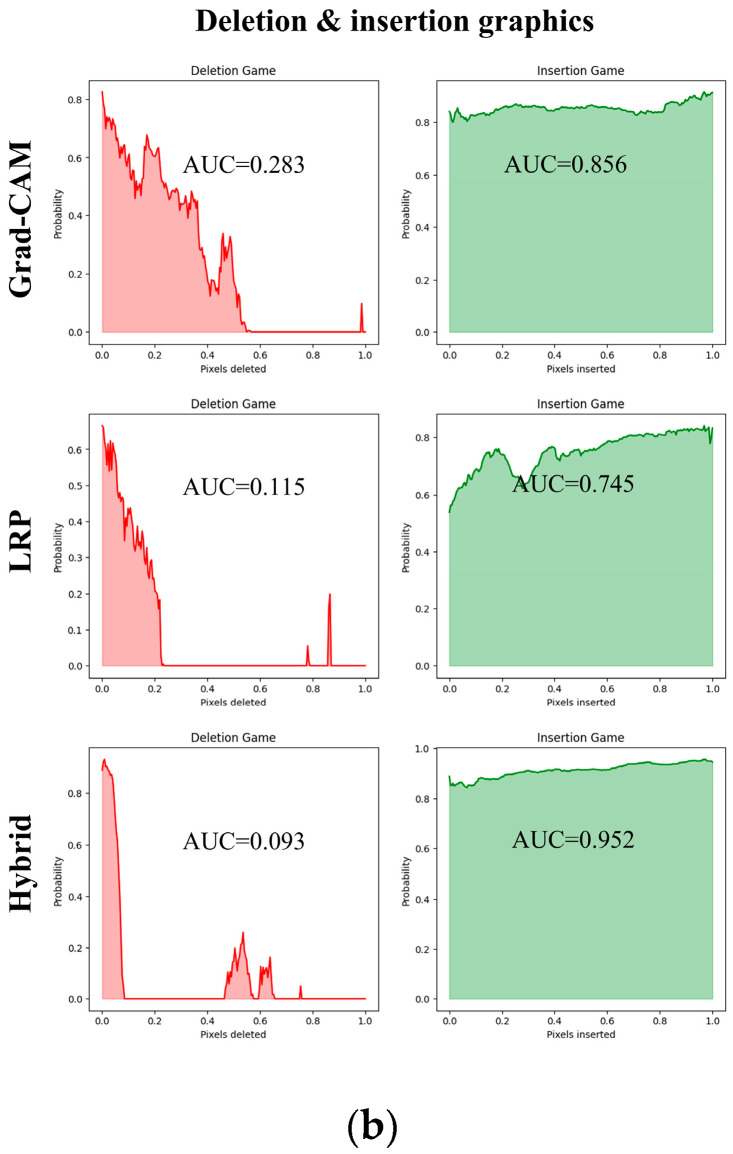
(**a**) Overlaid images obtained from Grad-CAM and LRP, along with their corresponding hybrid approach. (**b**) Deletion and insertion graphs corresponding to the visualizations.

Additionally, Figure 5a,b provide the deletion and insertion results for a sample image of monkeypox class, further emphasizing the superior performance of Grad-CAM and LRP.

**Figure 6:**
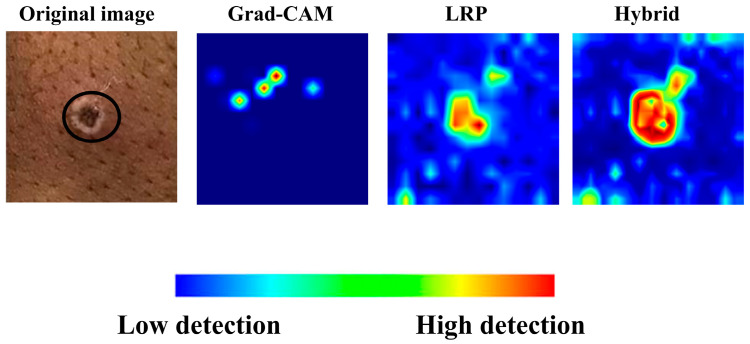
Grad-CAM, LRP, and their corresponding hybrid heatmap output.

In Figure 6, the heatmaps and hybrid output for another image in the monkeypox class, generated solely using LRP and Grad-CAM, are presented. The hybrid heatmap effectively highlights a single critical region of interest, demonstrating the combined strength of both methods in identifying the most discriminative features for accurate classification. This result underscores the ability of the hybrid approach to focus on highly specific and clinically relevant areas, which is particularly crucial for challenging cases such as monkeypox, where precise localization of key features can significantly impact diagnostic accuracy.

## 4. Discussion

In this study, we successfully fine-tuned Transformer-based models to classify monkeypox lesions against similar conditions such as measles and chickenpox in a classification framework. Our findings demonstrate not only reliable differential diagnosis but also substantial improvements in model interpretability. By integrating multiple XAI techniques including Grad-CAM, LRP, and AR and generating hybrid heatmaps via PCA, we provide quantitative and comprehensive visual explanations of the decision-making process.

The literature reveals two distinct trends in monkeypox detection studies. On one hand, several studies have enhanced model interpretability by incorporating XAI techniques such as LIME and Grad-CAM into CNN-based [20,22] and, to a lesser extent, Transformer-based frameworks [23]. Among these studies, the best accuracy for the MSLD was achieved by a CNN-based model, with a performance of 97.63%, while the Transformer-based model, utilizing augmented data, achieved an accuracy of 94.6%. Moreover, using the MSID, the highest accuracy of 98.19% was achieved with the attention-based model, and its predictions were interpreted using the same explainability techniques [17]. It should also be noted that the MSLD in the literature offers two versions: original and augmented. Generally, the majority of studies have utilized the augmented version. However, in this study, to avoid potential biased results from XAI methods, the original version of the data is used, and data augmentation is applied during the training phase. These studies enhance transparency by highlighting relevant regions but rely on qualitative heatmap assessments without robust quantitative validation. On the other hand, a considerable number of studies focus exclusively on classification performance, using deep learning models without any explainability [28,31]. Such approaches emphasize metrics like accuracy, precision, and recall but do not provide insights into the model’s decision-making process, thereby limiting clinical trust and understanding. Moreover, the existing monkeypox datasets in the literature are fundamentally based on the MSLD. The key distinction in the MSID is the inclusion of a “normal” class, which does not contain any lesions for diagnosis. As a result, applying explainability methods to this class would be meaningless. It is also important to highlight that publicly available datasets for monkeypox in the literature remain extremely limited. Overall, while existing literature has made significant progress in both performance and interpretability, there remains a critical gap: advanced Transformer-based classifiers have not been thoroughly integrated with innovative XAI methodologies that extend beyond traditional techniques, nor have the generated explanations been quantitatively validated. This gap underscores the need for research that systematically combines robust Transformer architectures with state-of-the-art and quantitatively assessed XAI methods to enhance both diagnostic accuracy and model transparency in monkeypox detection.

In the post-training phase, we evaluated the quality of the extracted features using visualization techniques such as t-SNE and UMAP. These analyses revealed that the ViT model forms more distinct and well-separated clusters compared to DeiT, indicating its superior ability to capture complex data structures and achieve effective class discrimination. Considering the results in Table 7 and Table 8, computed as averages across all test images, we quantitatively validate the superior ability of the hybrid method to preserve essential features while minimizing irrelevant ones. Although good average results are achieved across the entire test set, expected deletion and insertion values are not always observed in some individual images. Therefore, the number, quality, and diversity of the images emerge as key factors that can impact the outcomes in the dataset and may influence the results for generalization. Together, these findings underscore the robustness of the ViT model in both classification performance and interpretability for monkeypox detection, paving the way for more transparent and trustworthy AI-driven diagnostics.

The practical significance of this study lies in its potential to improve the interpretability and diagnostic reliability of Transformer-based classification models for monkeypox datasets. In our study, an effective training strategy was employed to address the challenge of training Transformers on small datasets. Since Transformers are typically require large-scale datasets, we fine-tuned pre-trained model weights along 50 epochs. During training, the best-performing fold was saved and utilized in the test phase, ensuring optimal model performance. Unlike other studies, several distinctive training strategies are implemented. Current augmentations are crucial for enhancing the model’s robustness. Given the limited dataset size, augmentations help the model better handle variations in real-world data, reducing the risk of overfitting.

Advanced techniques such as cut–mix, mix-up, label smoothing, and early stopping were incorporated. Cut–mix and mix-up encourage the model to learn more discriminative features by blending images and labels, which acts as an effective data augmentation strategy. Label smoothing helps mitigate overconfidence in predictions, smoothing out extreme probabilities and enhancing model calibration. Early stopping prevents overfitting by monitoring the validation loss, ensuring that the model does not continue training beyond its optimal performance. During fine-tuning, while 50 epochs were initially set, training for the MSID dataset was halted at epoch 40 to avoid overfitting as illustrated in Table 4. These strategies collectively contributed to improving the model’s capability to prevent overfitting due to the limited dataset, and optimizing performance during the training phase.

For the MSLD (Table 5), which contains two classes (Monkeypox vs. Others), ViT achieves perfect recall (1.0000) for the “Others” class, while maintaining a high precision (0.8750), resulting in a strong F1-score of 0.9333. This indicates that the model is able to correctly identify almost all “Others” samples without a substantial number of false positives. The Monkeypox class shows slightly lower recall (0.7895), suggesting some misclassifications; however, the precision remains perfect (1.0000), which is reflected in a balanced F1-score of 0.8824. Overall, ViT outperforms DeiT in average accuracy and AUC, demonstrating its superior capability in capturing relevant features even in limited-data scenarios. For the MSID (Table 6), which involves four classes (Monkeypox, Chickenpox, Measles, Normal), more nuanced patterns emerge. The Normal class is consistently well-separated, achieving very high recall and precision for both models, indicating that the model can easily distinguish normal skin samples from the three viral infections. In contrast, Chickenpox, Measles, and Monkeypox classes show overlapping patterns. For example, ViT attains a relatively lower precision for Chickenpox (0.5909) but a higher recall (0.8125), indicating that while many Chickenpox samples are correctly identified, some false positives are included from other classes. Conversely, Measles shows high recall (0.9333) but slightly lower precision (0.8235), reflecting confusion primarily with visually similar classes. These patterns highlight the challenges posed by inter-class similarity in multi-class settings and underscore the need for robust feature extraction methods, such as ViT, to resolve subtle distinctions. Comparing ViT and DeiT across both datasets, ViT consistently achieves higher average accuracy and AUC values. Class-wise differences, particularly in the multi-class MSID dataset, reveal that the choice of Transformer backbone affects the model’s ability to separate visually similar disease classes. Overall, these analyses suggest that ViT provides more discriminative feature representations, which are essential for accurate monkeypox diagnosis in both two-class and multi-class scenarios.

In our experiments with ViT, we integrated Grad-CAM, LRP, and AR methods for model interpretability. By hybridizing Grad-CAM and LRP images using PCA, we achieved the optimal deletion and insertion metrics. Notably, standalone LRP yielded the second-best results in these causal metrics, with a deletion score of 0.228 ± 0.017 and insertion score of 0.859 ± 0.013 on the MSLD dataset, and 0.219 ± 0.012 deletion and 0.888 ± 0.006 insertion on the MSID dataset. LRP distributes relevance more precisely across the feature maps by back-propagating importance scores layer-by-layer, which appears to align well with the self-attention mechanisms in ViT. This results in localized and faithful attributions that effectively capture the critical regions influencing the model’s predictions [63]. The superior performance of the hybrid approach likely stems from leveraging the complementary strengths of Grad-CAM, which provides coarse localization, and LRP, which offers precise Layer-Wise Relevance Propagation [64]. In contrast, the Rollout method consistently underperformed across test image sets, with a deletion score of 0.345 ± 0.032 and insertion score of 0.843 ± 0.026 on the MSLD, and 0.265 ± 0.018 deletion and 0.855 ± 0.014 insertion on the MSID. Rollout aggregates attention weights from multiple layers, potentially diluting the fine-grained relevance signals and introducing noise from less informative regions [58]. Consequently, this over-aggregation may lead to a loss of critical local details, thereby negatively affecting the causal metrics. These findings suggest that while LRP alone is effective, its integration with Grad-CAM via PCA significantly enhances interpretability by more accurately preserving essential features and minimizing irrelevant ones.

This study presents a comprehensive approach by integrating advanced XAI techniques with Transformer-based architectures for the detection of monkeypox, a challenging and less-explored domain. A major strength lies in the rigorous evaluation using both qualitative and quantitative assessment methods, including hybrid techniques to achieve the most reliable interpretability metrics. Unlike most existing studies that rely solely on classification performance or heatmap assessments, our work offers a deeper, quantitatively validated interpretability framework. However, some limitations should be acknowledged. First, the relatively small and imbalanced dataset may restrict the generalizability of the models even though extensive augmentation techniques are employed. Additionally, while our hybrid method outperformed, the complexity of combining heatmaps using PCA may pose challenges for real-world clinical deployment. Lastly, although our approach shows promising results, Transformers typically require large-scale datasets to avoid overfitting, which may limit their applicability in low-resource scenarios. This study improves monkeypox classification while introducing a robust, validated interpretability framework. The results highlight hybrid XAI’s potential to enhance the transparency and clinical reliability of Transformer-based models. In terms of clinical applicability, such a framework could be incorporated into electronic dermatology workflows as a decision-support tool, providing clinicians with interpretable outputs alongside predictions. Furthermore, the integration into telemedicine platforms could facilitate remote dermatological assessments, offering both accuracy and interpretability to enhance trust in AI-assisted care, especially in regions where access to specialists is limited.

## 5. Conclusions

In this study, we proposed a robust framework using fine-tuned ViT and DeiT models for classifying MSLD and MSID, achieving high performance through strategies that reduce overfitting. To improve interpretability, we applied LRP, Grad-CAM, and AR, and introduced a PCA-based hybrid method for more informative heatmaps. Quantitative evaluation with deletion and insertion metrics showed that Grad-CAM and LRP offer the most reliable visual explanations, supporting transparent and clinically meaningful decisions.

Future work can expand the dataset through multi-institutional collaborations and integrate clinical metadata to improve generalizability and performance. Additionally, by incorporating ground-truth lesion masks, future studies could compute Intersection over Union (IoU) to quantify the spatial alignment between XAI heatmaps and annotated lesions. Beyond IoU, these masks would also allow quantitative comparisons of saliency distributions within lesion versus non-lesion regions, as well as region-specific sensitivity analyses, thereby providing a more robust evaluation of explanation quality. Applying this approach to other dermatological conditions could further validate its broader applicability.

## Figures and Tables

**Table 1:** Augmented image samples for normal skin, measles, chickenpox, and monkeypox.

Images	Augmentation Operations
Normal	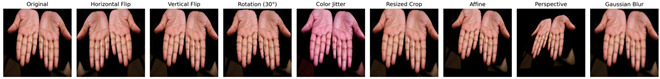
Measles	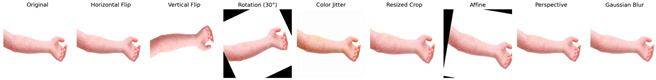
Chickenpox	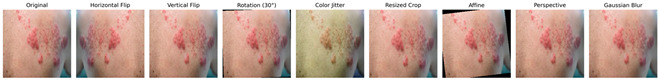
Monkeypox	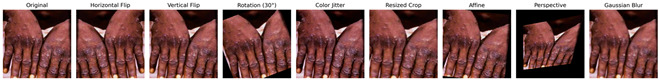

**Table 2:** Methods to prevent overfitting during the training stage.

Methods	Details of Hyper-Parameters	Numerical Values
Data augmentation	Horizontal flip Vertical flip	probability: 0.5
	Rotation	degrees: 30
	Color jitter	brightness: 0.2
		contrast: 0.2
		saturation: 0.2
		hue: 0.1
	Resized crop	scale: 0.8–1.0
	Affine	translate: 10%
		degrees: 10
		scale: 0.8–1.2
	Perspective	distortion scale: 0.5
		probability: 0.5
		interpolation: 3
	Gaussian blur	kernel size: 3
Regularization and optimization	Cut Mix/Mix Up	random choice
	Label smoothing	0.1
	Early stopping	15
	Learning rate	0.0001

**Table 3:** The graphs of training/validation accuracy and loss using ViT and DeiT for each epoch along with confusion matrix for MSLD.

	Train/Validation Accuracy	Train/Validation Loss	Confusion Matrix	ROC Curve with AUC
ViT	** 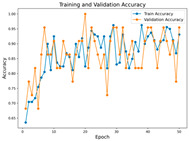 **	** 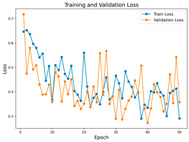 **	** 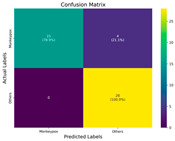 **	** 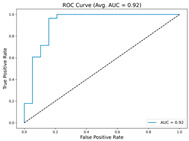 **
DeiT	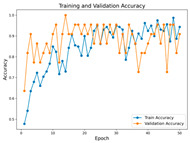	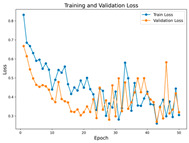	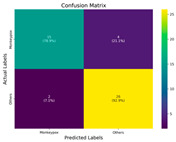	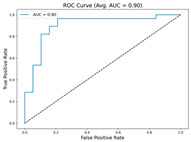

**Table 4:** Graphs of training/validation accuracy and loss using ViT and DeiT for each epoch along with confusion matrix for MSID.

	Train/Validation Accuracy	Train/Validation Loss	Confusion Matrix	ROC Curve with AUC
ViT	** 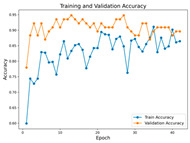 **	** 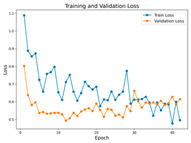 **	** 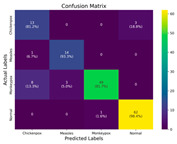 **	** 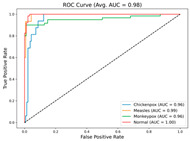 **
DeiT	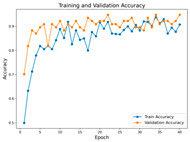	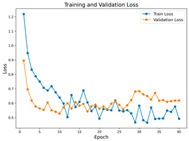	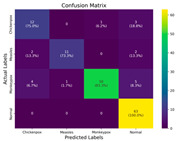	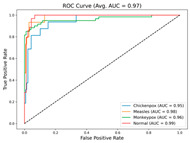

**Table 5:** The performance metrics obtained for MSLD with ViT and DeiT.

Transformer Method	Classes	Precision	Recall	F1-Score	Averaged Accuracy	AUC
ViT	Others	0.8750	1.0000	0.9333	0.9149	0.9192
	Monkeypox	1.000	0.7895	0.8824		
DeiT	Others	0.8667	0.9286	0.8966	0.8723	0.9004
	Monkeypox	0.8824	0.7895	0.8333		

**Table 6:** The performance metrics obtained for MSID with ViT and DeiT.

Transformer Method	Classes	Precision	Recall	F1-Score	Averaged Accuracy	AUC
ViT	Chickenpox	0.5909	0.8125	0.6842	0.8961	0.9784
	Measles	0.8235	0.9333	0.8750		
	Monkeypox	0.9800	0.8167	0.8909		
	Normal	0.9538	0.9841	0.9688		
DeiT	Chickenpox	0.6667	0.7500	0.7059	0.8831	0.9708
	Measles	0.9167	0.7333	0.8148		
	Monkeypox	0.9804	0.8333	0.9009		
	Normal	0.8630	1.000	0.9265		

**Table 7:** Averaged causal scores across test set in MSLD utilizing ViT classification model.

Causal Metrics	Grad-CAM	LRP	AR	Paired Hybrid (Grad-CAM + LRP)
Deletion	0.278 ± 0.002	0.228 ± 0.017	0.345 ± 0.032	0.209 ± 0.026
Insertion	0.842 ± 0.011	0.859 ± 0.013	0.843 ± 0.026	0.865 ± 0.005

**Table 8:** Averaged causal scores across test set in MSID utilizing ViT classification model.

Causal Metrics	Grad-CAM	LRP	AR	Paired Hybrid (Grad-CAM + LRP)
Deletion	0.238 ± 0.015	0.219 ± 0.012	0.265 ± 0.018	0.192 ± 0.010
Insertion	0.873 ± 0.009	0.888 ± 0.006	0.855 ± 0.014	0.899 ± 0.004

## Data Availability

The original data presented in the study are openly available in https://www.kaggle.com/datasets/dipuiucse/monkeypoxskinimagedataset, accessed on 7 September 2025.

## Abbreviations

The following abbreviations are used in this manuscript:

CNN	Convolutional Neural Network
ViT	Vision Transformer
DeiT	Data-Efficient Image Transformer
Grad-CAM	Gradient-weighted Class Activation Mapping
LRP	Layer-wise Relevance Propagation
AR	Attention Rollout
PCA	Principal Component Analysis
XAI	eXplainable Artificial Intelligence
MSID	Monkeypox Skin Images Dataset
MSLD	Monkeypox Skin Lesion Dataset
MID	Monkeypox Image dataset
LIME	Local Interpretable Model-agnostic Explanations
ML	Machine Learning
AUC	Area Under Curve
